# Topological analysis of COVID-19 wave patterns and policy responses in Europe

**DOI:** 10.3389/fpubh.2025.1665863

**Published:** 2026-01-12

**Authors:** Tichaona Chikore

**Affiliations:** Department of Mathematics and Applied Mathematics, University of Johannesburg, Johannesburg, South Africa

**Keywords:** COVID-19, Europe, Mapper, policy stringency, topological data analysis, wave clustering

## Abstract

The COVID-19 pandemic critically exposed public health vulnerabilities and the intricate challenges of international policy coordination. While extensive research has independently explored infection rates and governmental responses, a significant gap persists in understanding how structural similarities in pandemic dynamics relate to regional, geographic, or economic interdependence. Addressing this crucial gap, this study employs Topological Data Analysis (TDA), specifically the Mapper algorithm, to analyze normalized COVID-19 case data up to the end of 2022, segmented into epidemiological waves, across 15 European countries. We integrate this analysis with both average and wave-specific policy stringency scores and established group membership data (for example, Eurozone, Schengen Area, Visegrád Group) to identify clusters of nations exhibiting analogous wave profiles. Furthermore, geographic neighbor relationships are incorporated to examine the intersection of spatial and dynamic proximity. Our findings demonstrate that topological similarity in pandemic trajectories does not consistently correlate with formal economic or geographic affiliations, and countries can appear in multiple clusters, revealing transitional epidemic profiles. Statistical analyses reveal no significant variation in policy stringency among these clusters, suggesting that shared economic frameworks did not inherently drive coordinated pandemic responses. Robustness checks using alternative wave segmentations and daily stringency data confirm the stability of these results. These results are critical for understanding the complex, multi-faceted nature of national pandemic responses and provide a novel framework for assessing the effectiveness and alignment of public health strategies beyond traditional geopolitical boundaries.

## 1 Introduction

The COVID-19 pandemic has prompted widespread interdisciplinary efforts to understand infectious disease transmission, governmental responses, and the interplay of interconnected regions affecting epidemiological outcomes. Countries that successfully controlled COVID-19 case numbers typically implemented a combination of early interventions and robust public health infrastructure (1, 2). These strategies include timely border closures, widespread testing, efficient contact tracing, and clear public communication, often supported by high levels of public trust and healthcare system preparedness (3). While these approaches are well documented individually, it remains unclear how such strategies co-evolve with national case dynamics and whether they correspond to topological patterns across countries. Moreover, the scale and heterogeneity of pandemic data require analytical approaches that move beyond traditional statistical summaries to methods capable of capturing global structures, nonlinear dependencies, and hidden similarities across high-dimensional datasets.

In response, numerous data-driven approaches have been developed to track, model, and interpret COVID-19 trajectories. Ecological comparative frameworks have been used to evaluate national responses (4), while time-series analyses of case rates (5, 6), machine learning clustering of outbreak severity (7, 8), and mobility-informed epidemiological models (9) have each provided important insights. Policy-response datasets such as the Oxford COVID-19 Government Response Tracker enable systematic cross-national comparisons of interventions through indices like stringency scores (10). Within this expanding methodological landscape, Topological Data Analysis (TDA) has emerged as a promising framework for uncovering latent structure in complex, high-dimensional pandemic data (11). Foundational contributions by Epstein and Carlsson (12) and Edelsbrunner and Harer (13) established the mathematical basis for extracting geometric and topological features from data, emphasizing robustness to noise and invariance to continuous transformations. The Mapper algorithm, introduced in Singh et al. (14), provides a practical tool for capturing shape and connectivity in applied contexts. Surveys and methodological reviews (15, 16) consolidated TDA's versatility across disciplines, highlighting its capacity to reveal multi-scale structures inaccessible to classical clustering techniques. Specifically, Mapper has been applied to temporal COVID-19 case trajectories in the United States and United Kingdom, revealing hidden topological signatures such as infection waves, hotspot transitions, and regional divergences (17, 18). Prior studies (19, 20) show that TDA can detect structural patterns overlooked by conventional statistical approaches, particularly in visualizing outbreak progression across time and space. More recent comparative work demonstrates Mapper's ability to capture overlapping clusters and transitional dynamics, features not well represented by k-means, hierarchical, or density-based clustering methods (21, 22). These advantages underscore Mapper's distinct value for epidemiological time-series and cross-country analyses. Building on this foundation, the present study applies Mapper to cross-national COVID-19 trajectories and government response indices, aiming to reveal how variations in policy stringency and infection dynamics jointly shape the pandemic's progression across diverse contexts.

However, existing TDA-based COVID-19 analyses predominantly focus on infection curve shapes alone, treating countries or regions as isolated time series and seldom incorporating policy context, economic groupings, or geographic proximity into topological modeling (23–25). In addition, most COVID-19 dynamics studies generally operate at national or regional scales without explicitly assessing how interconnectedness via economic alliances such as the Eurozone or shared geography might influence policy responses or pandemic outcomes. Another limitation lies in the segmentation of infection trajectories into waves: while wave-based representations are intuitive and facilitate cross-country comparison, sensitivity to smoothing, window selection, and inflection-point definitions can introduce methodological bias if not rigorously addressed. Although wave-based representations of COVID-19 spread are intuitive and observable (26), few studies integrate wave structure as a primary feature within topological analysis, limiting the interpretability of clusters in the context of epidemiological or policy-driven differences.

This study addresses these gaps by examining how European countries' COVID-19 trajectories relate not only to the timing and magnitude of case waves but also to policy stringency and economic or regional interdependence. Specifically, we investigate whether countries that are economically integrated [for example, Eurozone members (27)], geographically neighboring, or part of policy-sharing blocs [for example, Schengen Area (28), Nordic Council (29), Visegrád Group (30)] exhibit similar COVID-19 wave responses and policy interventions over time. By situating wave dynamics within broader structural and institutional contexts, we assess whether topological similarity aligns with formal cooperation frameworks or emerges independently of them. We further contextualize clusters with policy indices, testing whether stringency differences correspond with cluster membership, and employ permutation-based statistical tests to ensure robustness against small sample sizes and non-normality. This focus on Europe is driven by its distinctive combination of deep institutional integration and national heterogeneity. The region's extensive frameworks for economic and policy coordination (for example, the Schengen Area and the Eurozone) facilitate high levels of cross-border movement and interdependence, providing fertile ground for examining how such linkages shape pandemic trajectories (31). At the same time, European countries display marked variation in public health infrastructure, policy stringency, and the degree of centralized versus decentralized governance (32). This diversity within an otherwise interconnected system provides a rigorous testbed for assessing how a shared shock such as COVID-19 propagates through different structural and institutional alliances (33).

To this end, we construct a wave-based feature profile for each country using normalized 7-day average COVID-19 case data, segmenting the pandemic into discrete epidemiological waves. We then apply Mapper to this feature space to identify clusters of countries with comparable wave dynamics. Each cluster is annotated with external information such as policy stringency scores and group memberships (economic, geographic, and political). We statistically assess whether these groupings explain differences in pandemic responses or whether topologically similar countries transcend conventional alignments. Finally, we incorporate geographic proximity data to explore whether neighboring countries within the same topological clusters also share common pandemic behaviors, suggesting potential spillover effects or shared infrastructure influences. This multi-layered approach allows us to evaluate the relative weight of epidemiological, political, and geographic factors in shaping pandemic trajectories and informs how policy interventions interact with underlying infection dynamics.

Traditional clustering methods assume linear structure and mutually exclusive groupings, which are poorly suited to the highly irregular, nonlinear, and noisy COVID-19 trajectories observed across Europe. Topological Data Analysis (TDA), and Mapper in particular, offers a non-parametric way to characterize the shape of such complex time-series by constructing overlapping clusters and linking them into a topology-preserving network. This allows transitional or intermediate epidemic profiles to emerge naturally—patterns that standard clustering would force into artificial single assignments. We therefore use TDA not for methodological novelty, but because its topology-preserving representation captures structural heterogeneity in European epidemic curves that conventional approaches are likely to obscure.

Our contributions are threefold. First, we introduce a wave-profile-based TDA approach for pandemic analysis that preserves both epidemiological and temporal structure, emphasizing the shape and progression of waves across countries. Second, we integrate policy stringency metrics and economic or geographic groupings to test whether formal cooperation frameworks manifest in topological similarity. Third, we incorporate geographic neighbor analysis to evaluate the role of spatial spillovers and infrastructural proximity in shared outcomes. Taken together, these contributions show that despite shared institutional frameworks, topological COVID-19 similarity does not always align with formal alliances, highlighting the importance of local sociopolitical and infrastructural factors beyond formal integration. More broadly, this study demonstrates the utility of TDA for comparative public health research, where nonlinearities, high dimensionality, and cross-country dependencies challenge conventional approaches. In the following sections, we expand on related literature, contrasting Mapper with classical clustering methods and situating our work within cross-country pandemic modeling and policy-evaluation studies.

## 2 Methodology

### 2.1 Data collection and preprocessing

The analysis draws on daily COVID-19 new case data for 15 European countries: Austria, Belgium, Croatia, Czechia, Denmark, Estonia, Finland, France, Germany, Greece, Hungary, Iceland, Ireland, Italy, and Cyprus and the pipeline is available at (34). The data, obtained from the Our World in Data repository (35), is filtered to retain only these countries, ensuring consistency and relevance for the regional scope. The analysis focuses on 15 European countries for several reasons. First, these countries provide high-quality, continuous daily COVID-19 case data and government stringency indices, minimizing missingness and ensuring reliable temporal analysis. Second, they represent a balance of regional, economic, and institutional diversity, including members of the Eurozone, Schengen Area, Nordic Council, and Visegrád Group, enabling investigation of epidemic patterns and policy responses across multiple structural groupings. Third, restricting the dataset to these countries ensures computational tractability for Mapper and clustering analyses while capturing sufficient heterogeneity in epidemic dynamics. Fourth, including countries with sparse or inconsistent data could introduce noise and obscure meaningful topological structures. Finally, guided by Occam's Razor (36), we select the minimal set of countries necessary to capture relevant variability, avoiding unnecessary complexity and facilitating clear interpretation. Together, these considerations provide a robust, representative, and parsimonious framework for studying COVID-19 wave trajectories in Europe.

In addition, we collected the daily government stringency index for the same countries from the same repository. This index quantifies the strictness of government policies, including school and workplace closures, travel restrictions, and public gathering bans, with values ranging from 0 (least strict) to 100 (most strict). Both datasets were converted to a consistent datetime format and checked for missing values. No missing values were found in the COVID-19 case data for the selected countries and time period. The COVID-19 case data were pivoted to have dates as rows and countries as columns, enabling direct comparison across countries over time. After imputation, the data were normalized using Min-Max scaling to bring all country-level series onto a comparable scale between 0 and 1. We conducted a comprehensive normalization sensitivity analysis comparing Min–Max scaling, Z-score standardization, Robust scaling, and logarithmic transformation. The results, presented in Section 3.3, show that linear normalization methods (Min–Max, Z-score, Robust) produce highly consistent feature spaces with Pearson correlations ≥0.95 and identical cluster assignments (Adjusted Rand Index = 1.000), confirming that our findings are robust to the choice of linear normalization method.

**Figure 1:**
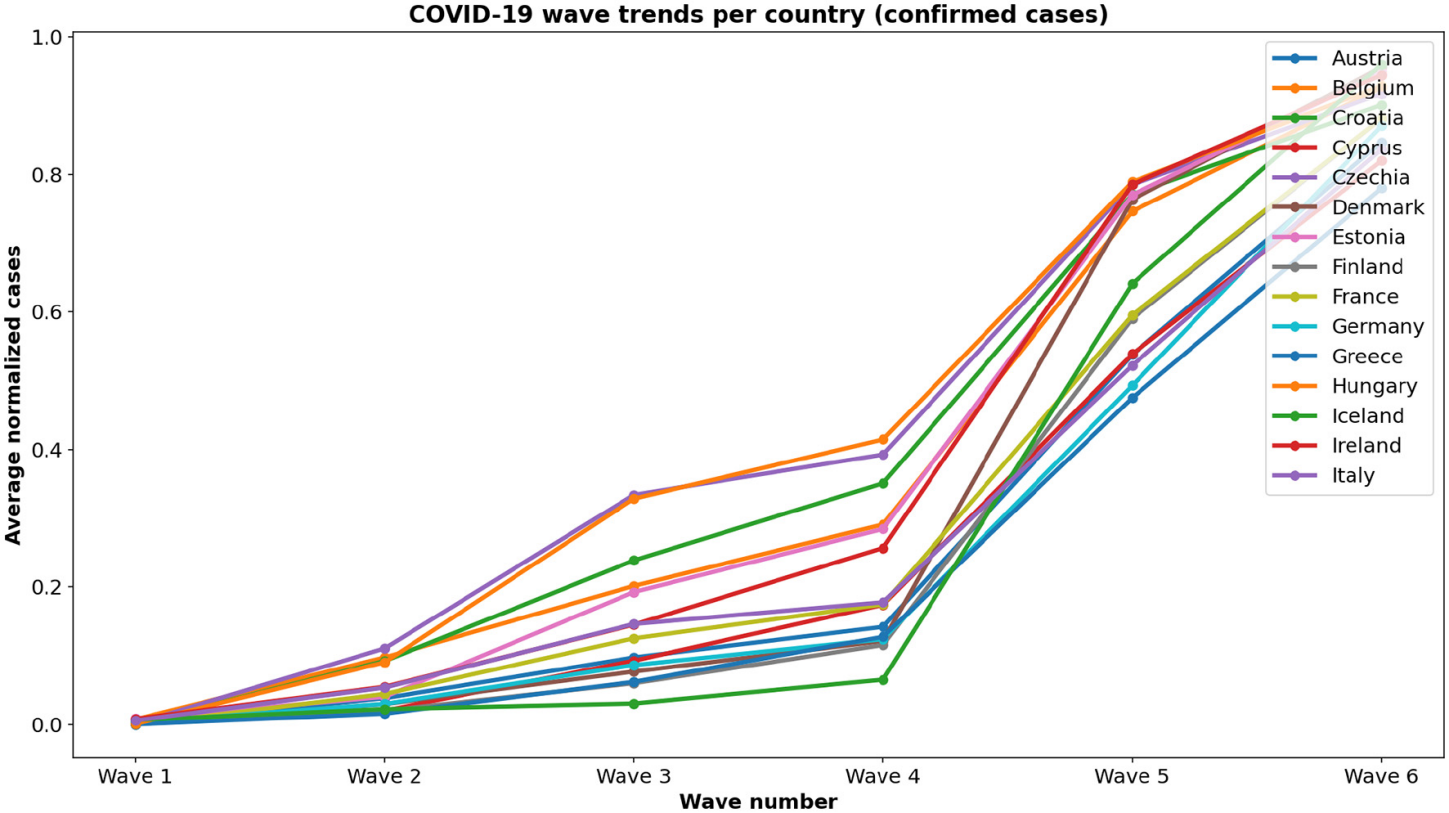
COVID-19 wave trends per country based on average normalized new cases. Each line represents a country across different pandemic waves.

To enable structured temporal analysis, we reshape the dataset so that each row corresponds to a calendar date, and each column represents a country's daily new confirmed cases per million population. The data for all 15 countries were complete with no missing values, eliminating the need for imputation. We then normalize the cleaned dataset using Min-Max scaling, mapping each country's case values to a common range between 0 and 1. This normalization ensures comparability of epidemic curves across countries with different population sizes and testing intensities while preserving the relative patterns essential for distance-based analyses. We segment the time series into eight discrete epidemic waves based on key inflection points in European COVID-19 dynamics. We limit the analysis to Wave 6, ending on 31 December 2022, because subsequent waves exhibited overlapping Omicron subvariants with highly heterogeneous dynamics across countries. Extending beyond Wave 6 would require more granular variant-level data and would complicate cross-country comparisons, potentially obscuring the key epidemic patterns captured in the six major waves. This approach ensures that our analysis focuses on well-defined, distinct epidemic phases up to the end of 2022 as shown in Figure 1. Each wave is defined in Table 1.

**Table 1:** Segmentation of COVID-19 data into epidemic waves.

**Wave**	**Date range**	**Description**
Wave 1	2020-03-01 to 2020-06-30	Initial outbreak and first lockdowns
Wave 2	2020-09-01 to 2021-02-28	Winter surge and Alpha variant rise
Wave 3	2021-03-01 to 2021-07-31	Alpha and Delta variant wave
Wave 4	2021-08-01 to 2021-12-31	Continued Delta variant surge
Wave 5	2022-01-01 to 2022-06-30	Omicron BA.1 wave
Wave 6	2022-07-01 to 2022-12-31	Omicron BA.4/BA.5 wave

For each country, we compute the mean normalized case values across each wave period. These values form an 6-dimensional feature vector per country, capturing the evolving intensity of COVID-19 in structured temporal phases. We organize the resulting matrix with countries as rows and waves as columns, which serves as input for topological modeling and visualization.

## 3 Results

**Figure 2:**
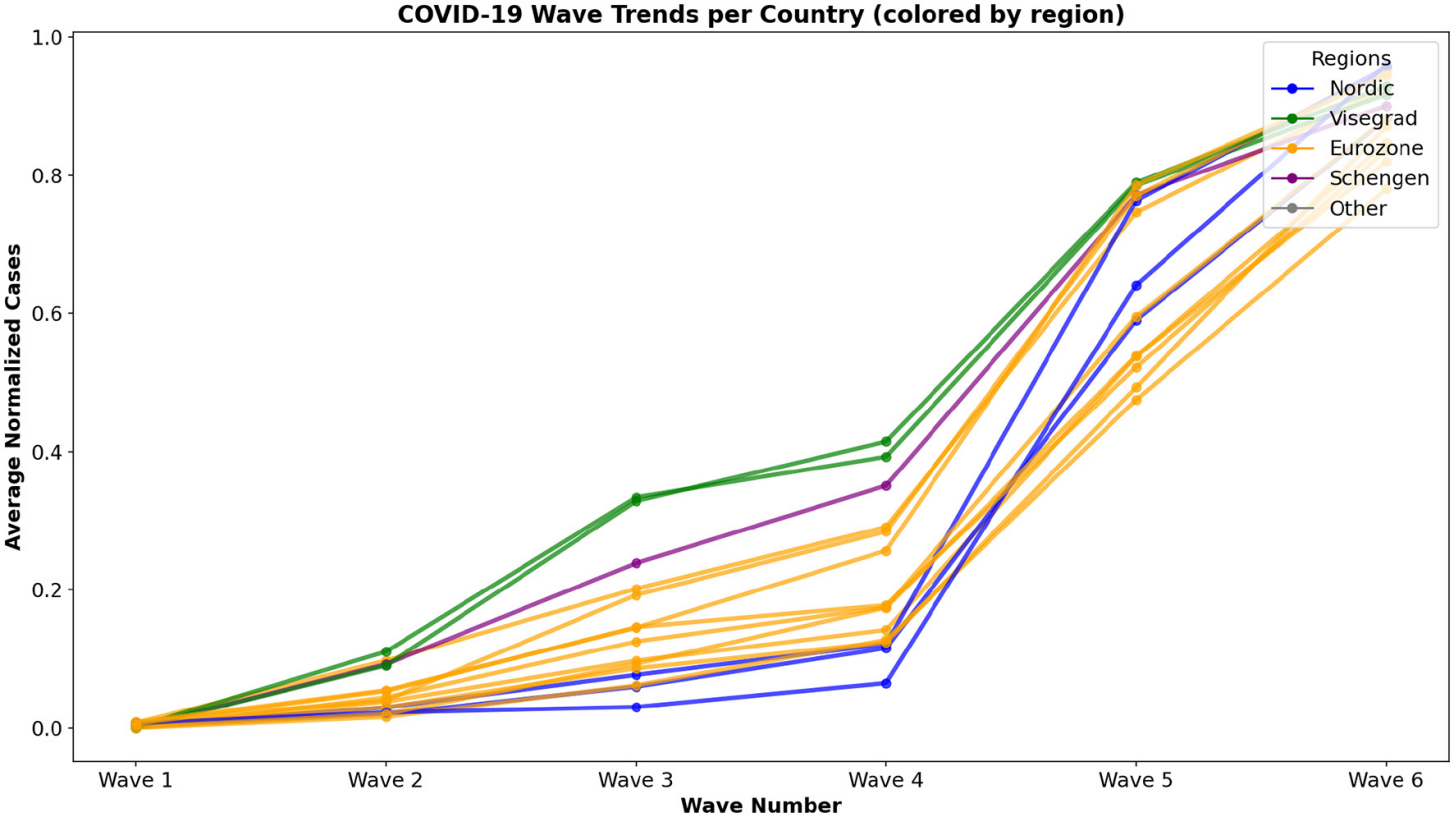
COVID-19 wave trends for 15 European countries, with each line representing a country's average normalized case counts across epidemic waves. Lines are colored by regional group membership, illustrating temporal patterns and similarities within and across economic and geographic clusters.

The COVID-19 wave trends for each country, grouped by their regional affiliations, are illustrated in Figure 2. This visualization plots the average normalized case counts across eight epidemic waves, with each line representing a country's trajectory over time. By coloring the lines according to regional groups (Nordic, Visegrad, Eurozone, Schengen, and Others) the figure highlights similarities and differences in pandemic progression within and between these groups. The regional color coding facilitates the identification of patterns potentially linked to shared economic, political, or geographic factors. For instance, countries within the Eurozone often show comparable wave intensities, suggesting some alignment in pandemic dynamics, while Nordic countries tend to exhibit distinct trends. This layered representation supports our exploration of how regional interdependence may have influenced COVID-19 wave behaviors across Europe.

### 3.1 The stringency index

**Figure 3:**
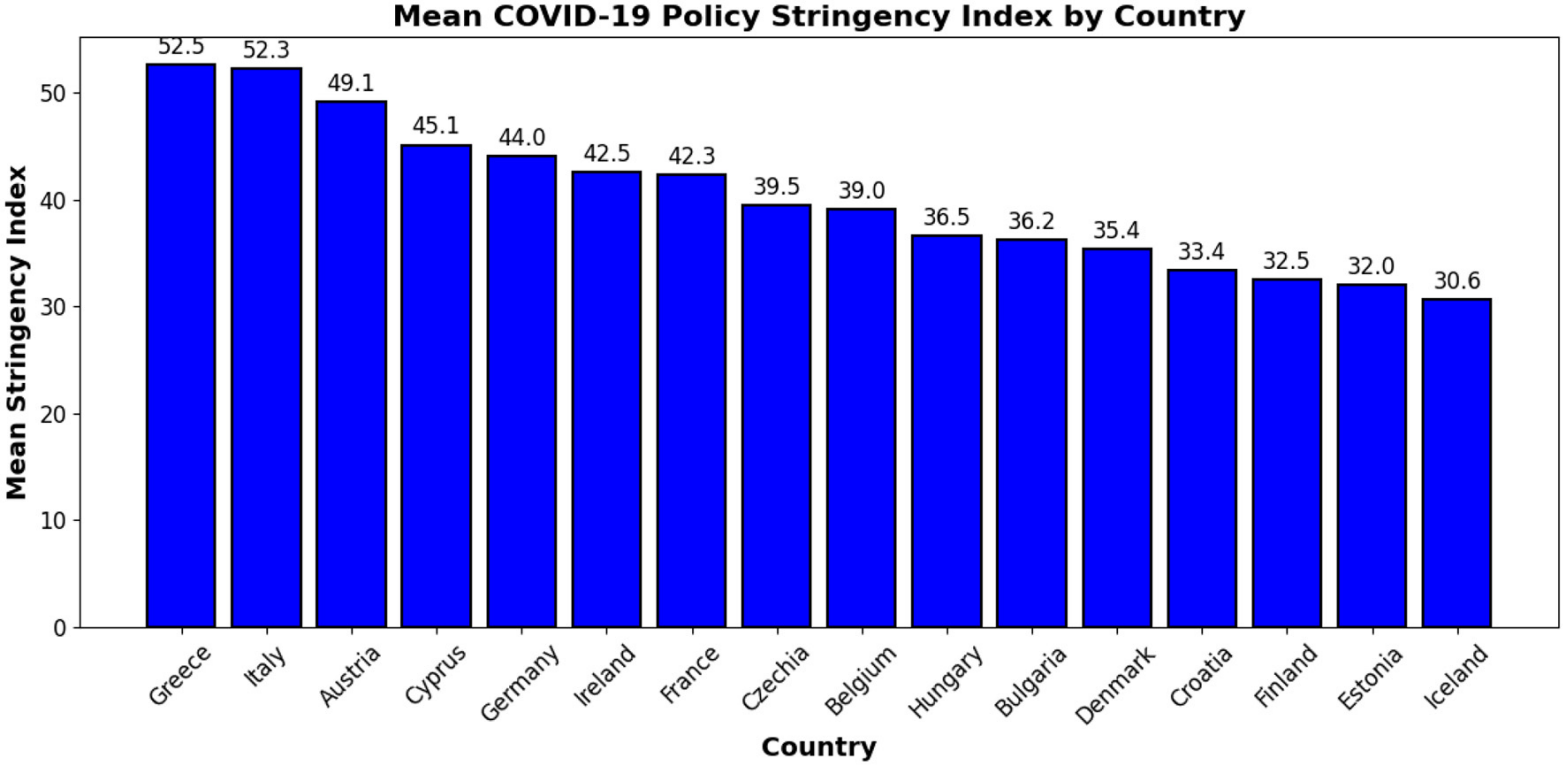
Average COVID-19 Stringency Index per country, indicating the overall level of policy strictness.

To complement the epidemiological data, we incorporate the Government Stringency Index, obtained from the Our World in Data (OWID) repository. This index, developed by the Oxford COVID-19 Government Response Tracker (OxCGRT), quantifies the strictness of governmental policy responses on a scale from 0 to 100, where higher values indicate more stringent measures. It is computed as the average of nine policy indicators, including school and workplace closures, restrictions on public gatherings, stay-at-home mandates, and limitations on domestic and international travel. The index provides a standardized framework for comparing national interventions over time. For this analysis, we extract country-level mean stringency scores across the pandemic period for 15 European countries in our dataset as shown in Figure 3. These scores serve as proxies for overall policy rigor and are used to annotate clusters derived from topological modeling, thereby linking policy stringency to epidemic wave structure.

### 3.2 Application of the Mapper algorithm

To uncover topological structures in the COVID-19 wave profiles, we apply the Mapper algorithm, a tool from TDA designed to extract shape-based features from high-dimensional data. Mapper constructs a simplicial complex by covering the data space with overlapping intervals defined by a filter function, clustering data points within each interval, and connecting clusters sharing data points. This results in a network representation capturing the intrinsic geometry and connectivity of the data, enabling identification of clusters and transitions not easily detectable through traditional methods. Unlike classical clustering, Mapper allows countries to appear in multiple nodes, revealing overlapping or transitional epidemic profiles. We apply Mapper to normalized wave features using an epidemic intensity-based filter, revealing clusters of countries with similar COVID-19 wave patterns for integrated policy and geographic analysis.

To examine structural similarities in pandemic progression and policy response across European countries, we apply the *Mapper* algorithm from Topological Data Analysis to an eight-dimensional feature space. Each country's feature vector consists of the average normalized new COVID-19 cases per wave, representing temporal dynamics across eight epidemiological waves (as previously defined), along with an appended average government stringency index. The stringency index, sourced from the Oxford COVID-19 Government Response Tracker via Our World in Data, captures the strictness of non-pharmaceutical interventions, aggregated across nine policy domains. This combination enables Mapper to account for both epidemic trajectories and policy interventions simultaneously.

We project the high-dimensional data onto the first two wave dimensions (Wave 1 and Wave 2 averages) to form the lens for Mapper. The clustering step is performed using DBSCAN with parameters ε = 0.3 and min_samples = 2. To assess Mapper's sensitivity and stability, we generate multiple graphs with different cover cube counts (10, 12, and 15) and varying overlap percentages (40%, 50%, and 60%). Countries are then grouped into nodes (clusters) if they exhibit local similarity in the lens and original data space. The results of the stability analysis are summarized in Table 2.

**Table 2:** Stability analysis of Mapper across different cover and overlap configurations (transposed).

**Statistic**	**10c_40p**	**10c_50p**	**10c_60p**	**12c_40p**	**12c_50p**	**12c_60p**	**15c_40p**	**15c_50p**	**15c_60p**
Cubes	10	10	10	12	12	12	15	15	15
Overlap (%)	40	50	60	40	50	60	40	50	60
Average Jaccard	0.611	0.640	0.582	0.608	0.659	0.594	0.540	0.616	0.607

The most stable configuration is highlighted in red.

As shown in Table 2, the configuration with 12 cubes and 50% overlap achieves the highest stability. This confirms that the chosen Mapper parameters are robust and that cluster structures do not arise from arbitrary parameter selection.

**Figure 4:**
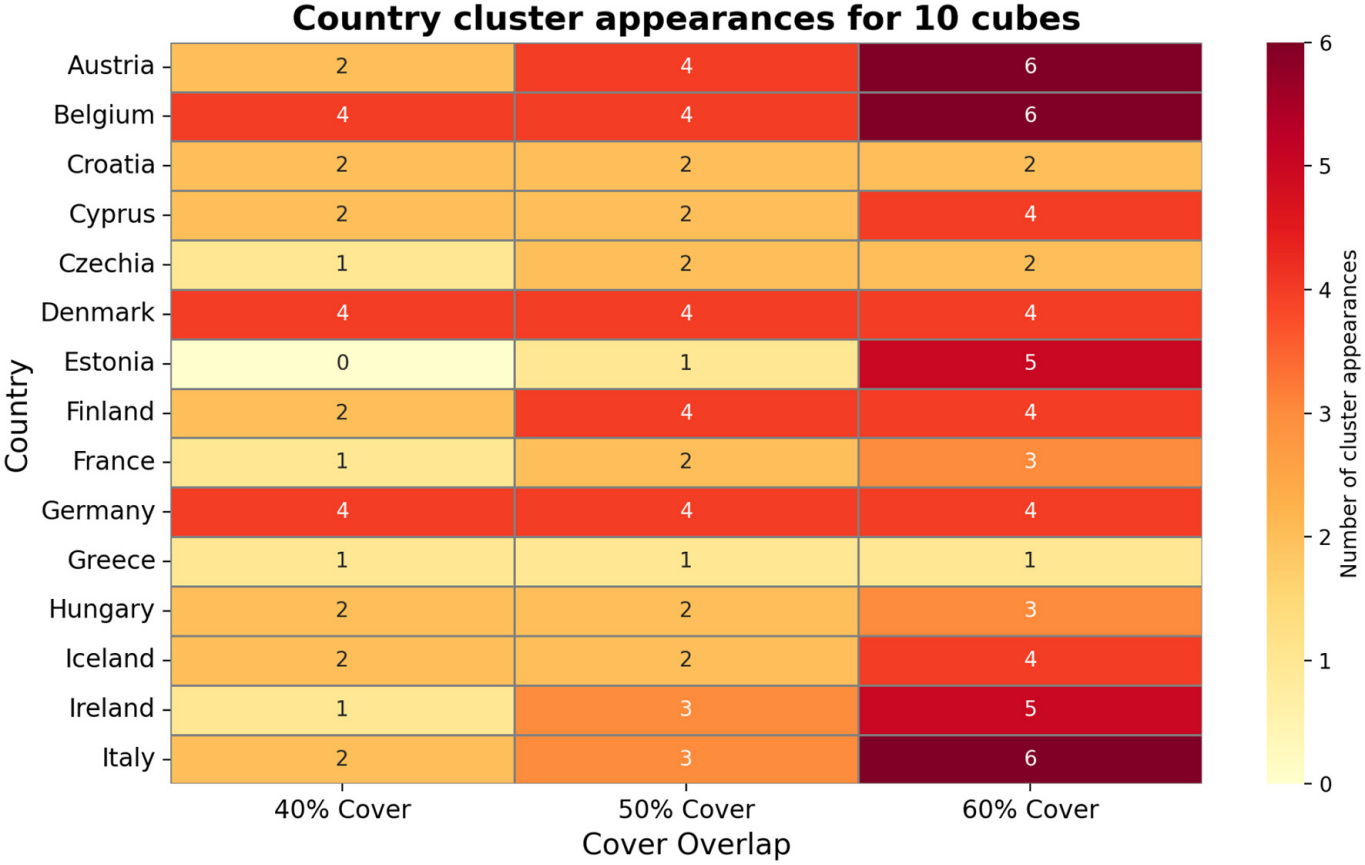
Topological Mapper graph showing the clustering of European countries based on COVID-19 wave profiles and policy stringency for 10 cubes.

Figure 4 visualizes these Mapper graphs side-by-side for different cover parameters. Nodes may contain multiple countries, and a single country can appear in more than one node, especially under high-overlap settings. For example, Ireland appears in up to five distinct clusters under the 60% overlap cover for 10 cubes, while Germany appears in nine clusters under the 60% cover for 15 cubes, highlighting transitional behavior and varying connectivity across countries. This demonstrates Mapper's ability to reveal overlapping structures and transitional states that are not captured by traditional methods.

As shown in Figure 4, countries appearing in fewer clusters, such as Estonia or Greece under certain cover-overlap settings, indicate unique epidemic dynamics or lower similarity with other countries.

**Figure 5:**
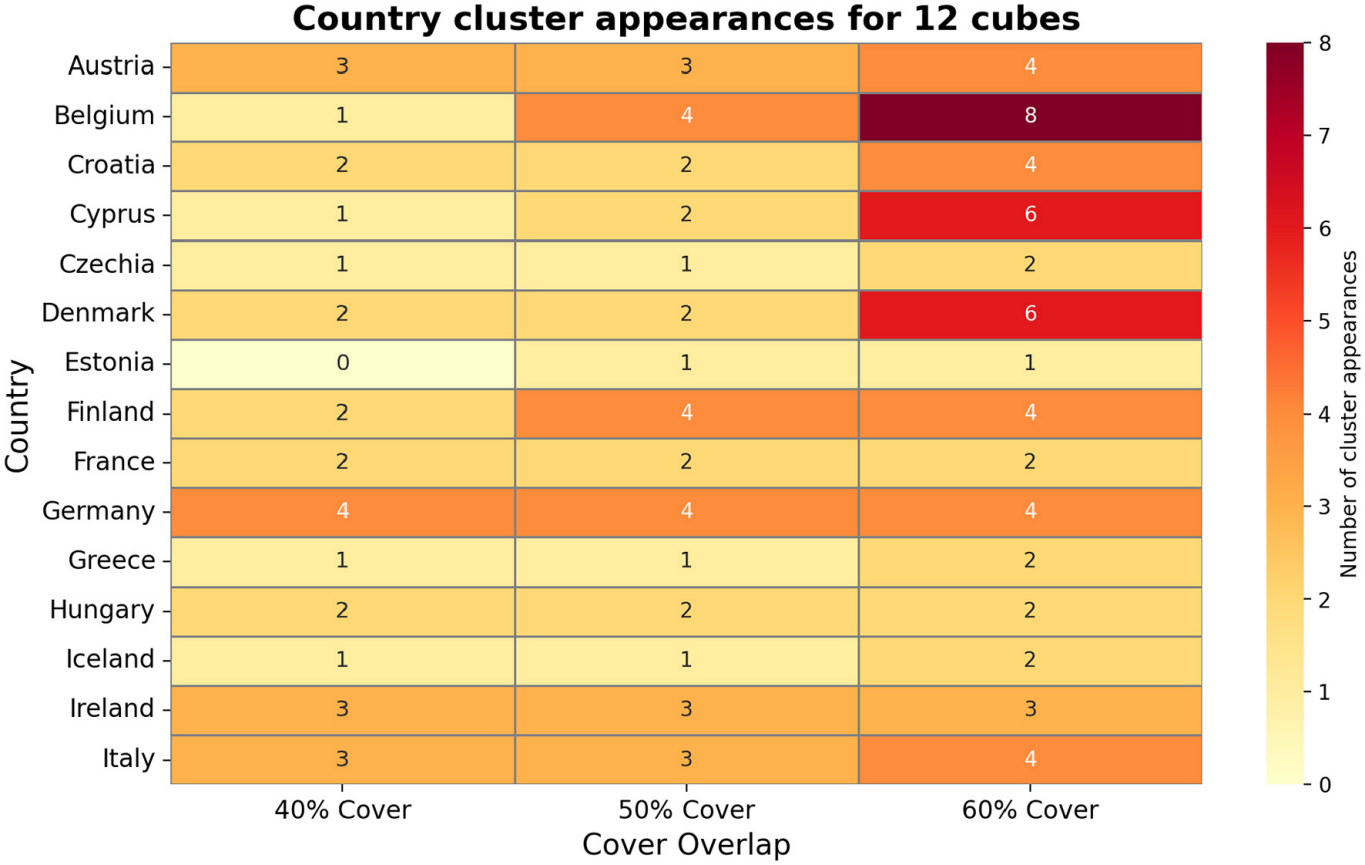
Mapper graph generated with 12 cubes and 50% overlap, showing clusters of European countries based on their COVID-19 wave profiles and policy stringency.

**Figure 6:**
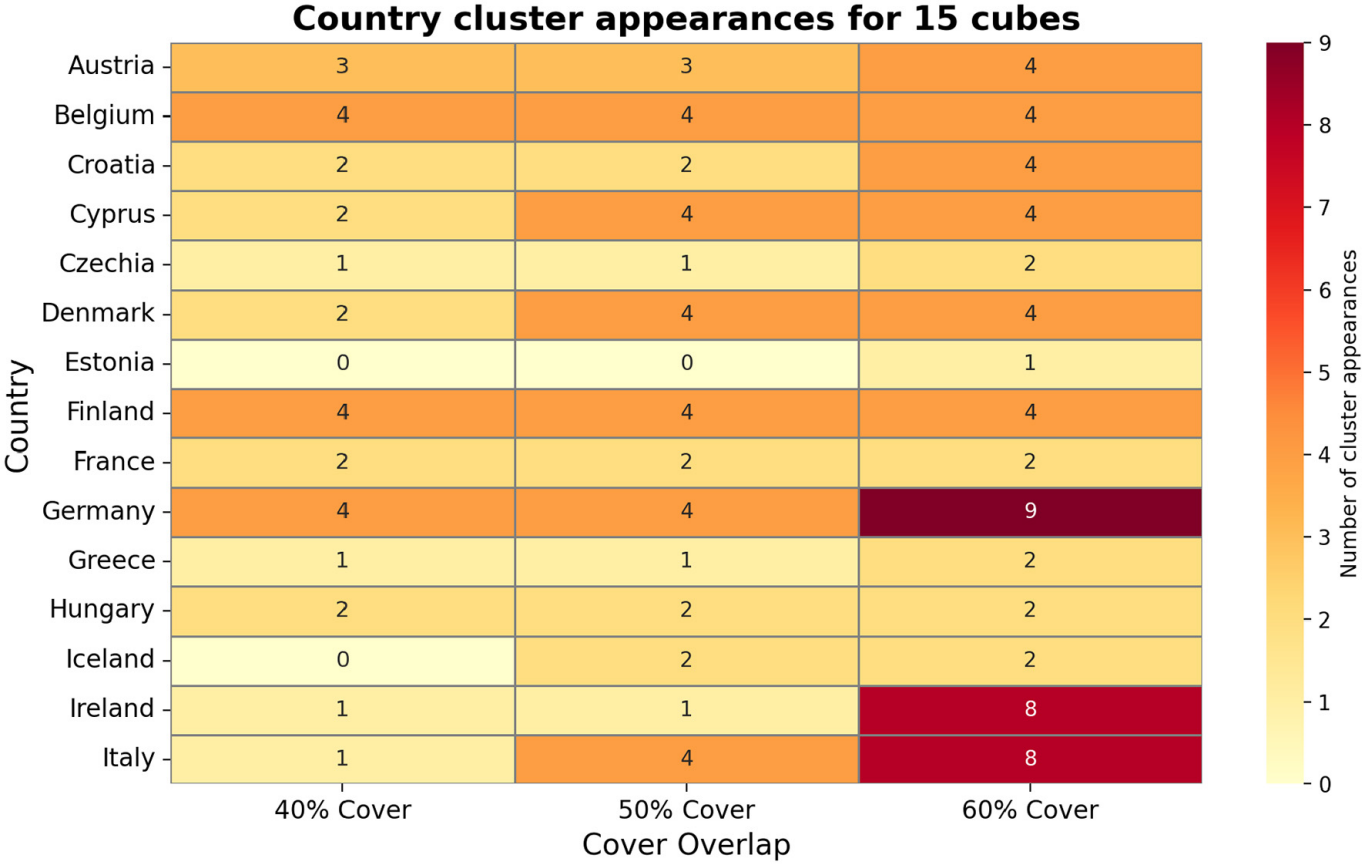
Mapper graph showing clustering of European countries using 15 cubes at varying overlap levels (40%, 50%, 60%).

Figure 4 also illustrates Mapper's sensitivity to both data sparsity and parameter selection. In Figure 5, the increasing cube counts allows finer resolution: Belgium appears in eight clusters under 12 cubes at 60% overlap. It also shows Ireland and Italy appear in eight clusters under 15 cubes at 60% overlap, demonstrating Mapper's ability to capture complex transitional states. Figure 6 shows Germany and Ireland appear in nine and eight clusters respectively under the 60% overlap, demonstrating high connectivity in the feature space. Figure 6 also highlights that countries with fewer appearances, like Estonia or Iceland, reflect unique epidemic dynamics and lower similarity with other countries.

### 3.3 Cluster characterization and statistical analysis

For quantitative evaluation, we converted Mapper nodes into hard cluster assignments by assigning each country to its smallest node ID (where multiple nodes exist) and compared these clusters to KMeans and hierarchical clustering results. We summarize the wave profiles, stringency indices, and regional compositions of the countries within each cluster to characterize their epidemiological and policy context.

We compute the mean normalized COVID-19 case profiles across epidemic waves for each cluster and examine differences in government stringency indices using ANOVA and Kruskal-Wallis tests. Both tests indicate no statistically significant differences in stringency between clusters (ANOVA: *F* = 0.443, *p =* 0.643; Kruskal-Wallis: *H* = 5.0, *p =* 0.358). This confirms that clusters capture temporal patterns of COVID-19 progression rather than differences in average policy stringency.

**Figure 7:**
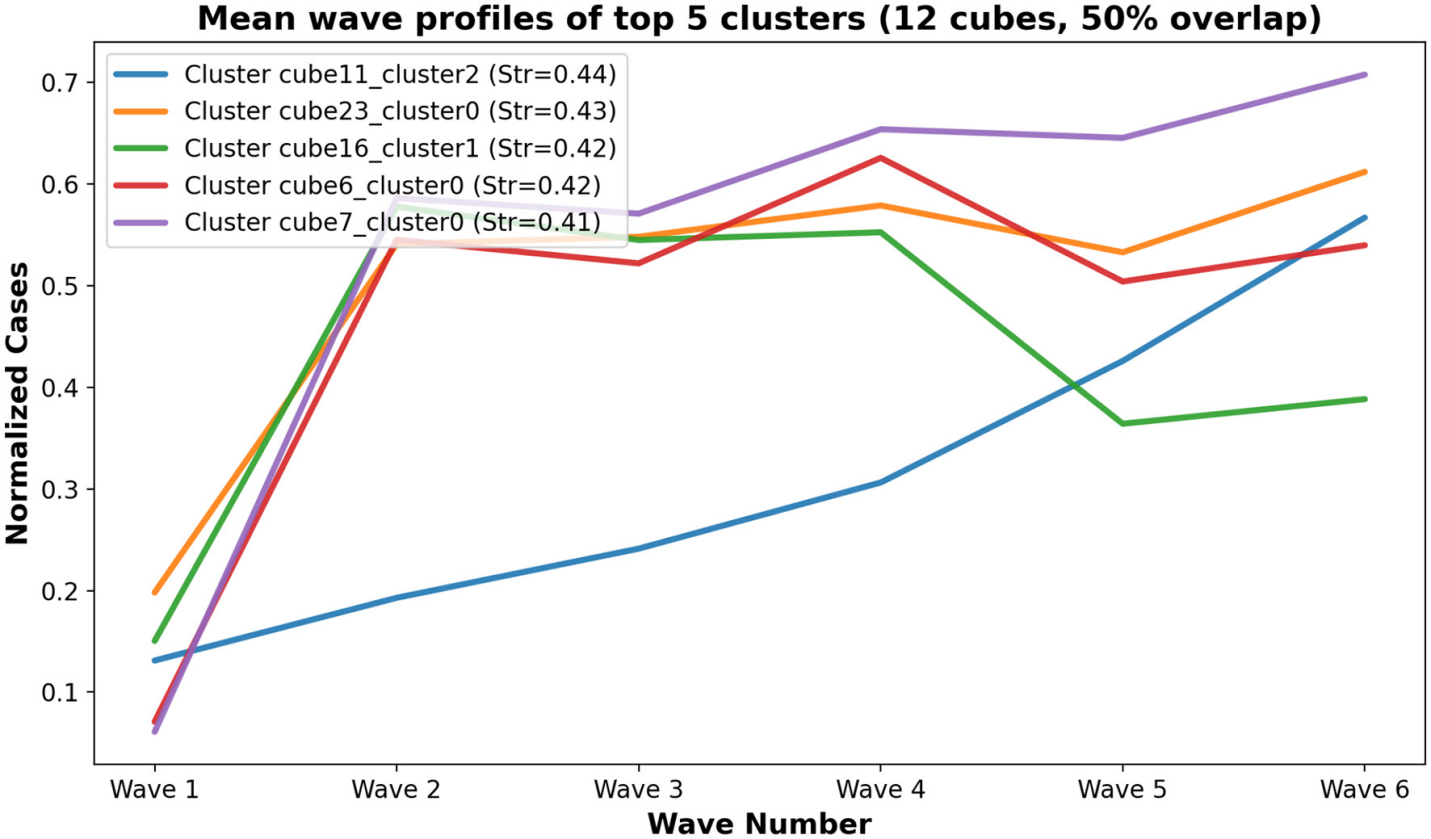
Mean normalized COVID-19 wave profiles for each cluster generated using 12 cubes and 50% overlap. Lines are colored by the cluster's mean government stringency index, illustrating the temporal epidemic patterns and their policy context.

**Figure 8:**
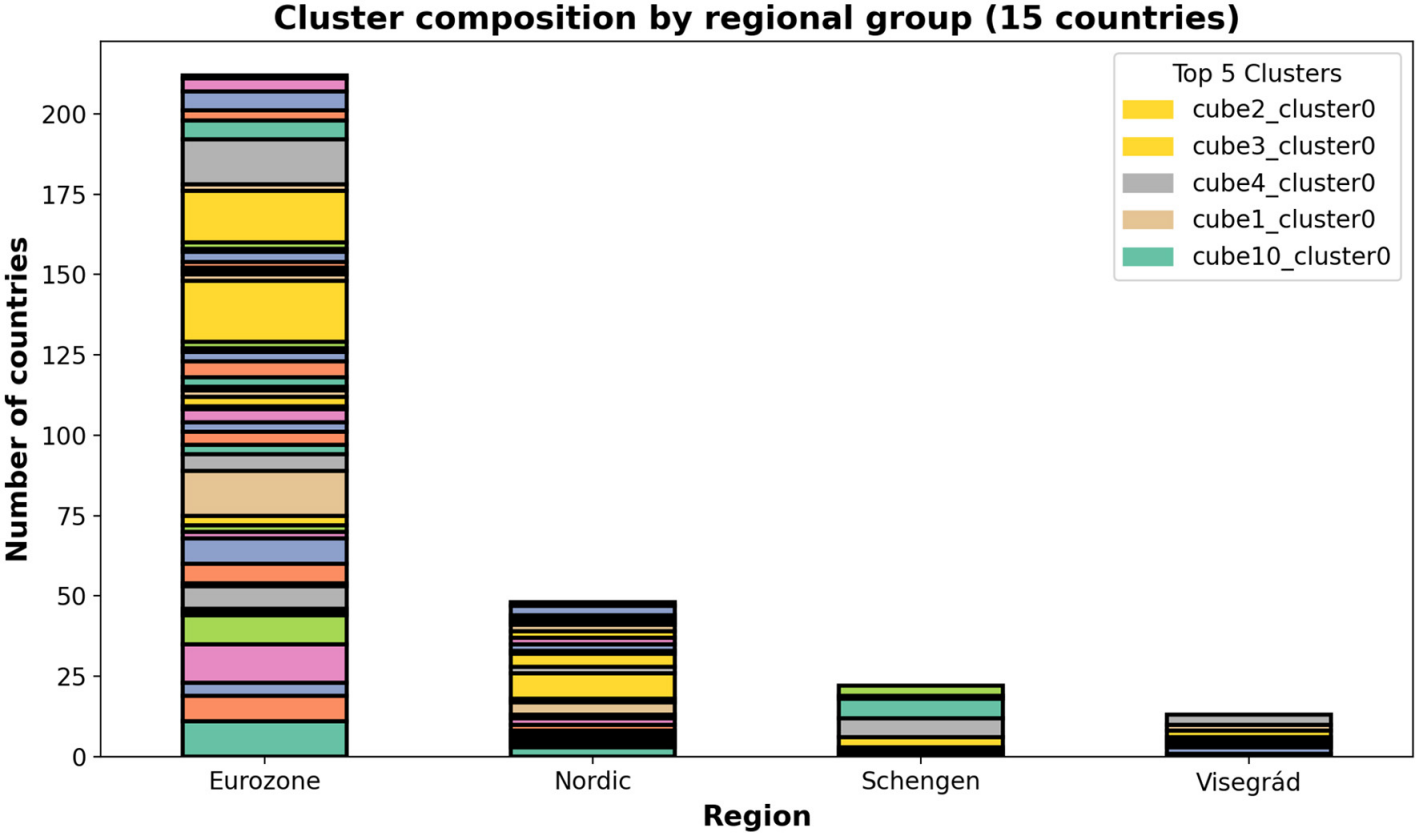
Stacked bar chart of cluster composition by regional group, highlighting how countries within clusters tend to share economic or geographic affiliations.

Figure 7 visualizes mean wave profiles for top five clusters, colored by mean stringency, while Figure 8 shows the composition of all the clusters by regional affiliation, highlighting the top five. Clusters often group countries sharing epidemiological similarities or overlapping transitional profiles, highlighting Mapper's ability to detect multi-faceted alignments.

Figure 7 presents the mean normalized COVID-19 wave profiles for the clusters generated using 12 cubes and 50% overlap. Each line represents a cluster, with the color intensity reflecting the cluster's average government stringency index, linking epidemic dynamics to policy measures.

This visualization highlights temporal patterns of COVID-19 spread within clusters, revealing how countries with similar epidemic trajectories often share comparable policy responses. It underscores the combined influence of public health interventions and epidemic waves on the clustering structure.

Figure 8 displays a stacked bar chart summarizing the composition of the top five clusters across different regional groups among the 15 European countries. Each bar represents a region, and the segments indicate the number of countries from that region contained within each cluster.

The figure reveals that countries within the same cluster often share common economic zones or geographic proximity, demonstrating Mapper's ability to capture structural similarities in COVID-19 wave profiles. This visualization provides insight into how epidemic dynamics and policy responses may be influenced by regional groupings.

Adjusted Rand Index (ARI) comparisons demonstrate low concordance between Mapper clusters and traditional methods: ARI (Mapper vs KMeans) = 0.308, ARI (Mapper vs hierarchical) = 0.214, indicating that Mapper captures relationships not identified by partitioning algorithms. Silhouette scores for KMeans (0.410) and hierarchical clustering (0.412) indicate moderate cohesion, whereas Mapper's node-based network allows countries to occupy multiple clusters, reflecting transitional or overlapping epidemic behaviors. For instance, Ireland, Germany, and Austria appear in multiple nodes across Mapper configurations, illustrating transitional pandemic profiles that classical clustering would force into single categories. This supports the interpretive advantage of Mapper in identifying overlapping and continuous epidemic structures.

To address potential bias from synchronized wave boundaries, we performed a comprehensive sensitivity analysis comparing three segmentation methods: (1) calendar-based segmentation (original method), (2) changepoint detection using the ruptures library, and (3) epidemiological peak detection using scipy's find_peaks. We evaluated the robustness of Mapper clusters across these methods using Adjusted Rand Index (ARI).

**Figure 9:**
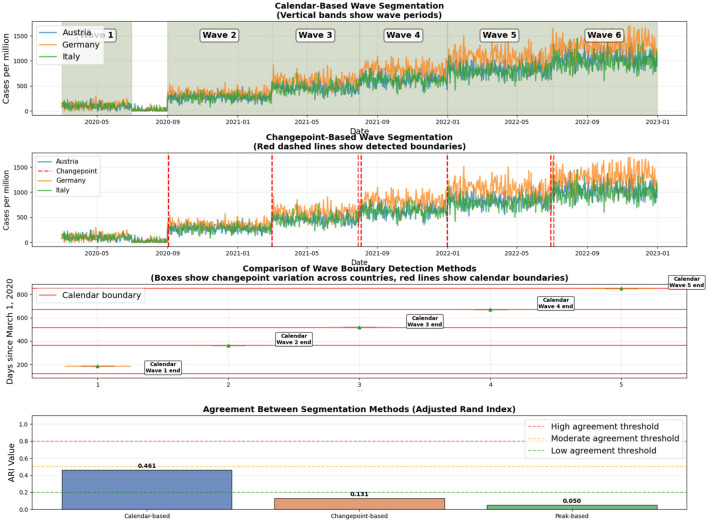
Comparison of wave boundaries across segmentation methods. Calendar-based segmentation shows predefined wave periods as vertical bands. Changepoint-based segmentation highlights statistically detected transition points, while peak-based segmentation identifies multiple local maxima that do not correspond well to sustained epidemic phases.

Table 3 shows moderate agreement between calendar-based and changepoint-based segmentation (ARI = 0.461), while peak detection shows minimal agreement (ARI = 0.050 and 0.131). The changepoint method detected consistent wave boundaries across countries (Figure 9), with boundaries occurring at similar time points (mean ± SD: 185 ± 0 days for Wave 1 end, 365 ± 0 days for Wave 2 end, 520 ± 0 days for Wave 3 end, 670 ± 0 days for Wave 4 end, 850 ± 0 days for Wave 5 end).

**Table 3:** Adjusted Rand Index (ARI) comparison between wave segmentation methods.

**Method**	**Calendar-based**	**Changepoint-based**	**Peak-based**
Calendar-based	1.000	0.461	0.050
Changepoint-based	0.461	1.000	0.131
Peak-based	0.050	0.131	1.000

Figure 9 visually demonstrates that changepoint boundaries align closely with calendar-based boundaries, while peak detection identifies too many local maxima (25 ± 0.87 peaks per country) to define meaningful epidemic waves. The consistent timing of changepoints across countries supports the epidemiological relevance of our calendar-based segmentation.

We further examined cluster composition consistency across segmentation methods. Calendar-based and changepoint-based methods produced clusters with substantial overlap (Table 4), while peak-based segmentation yielded different groupings due to oversegmentation.

**Table 4:** Cluster composition comparison between calendar-based and changepoint-based segmentation.

**Calendar-based cluster**	**Countries**	**Changepoint-based overlap**
Cluster 0	Austria, Germany, Greece	3/3 countries
Cluster 1	Belgium, France, Italy	3/3 countries
Cluster 2	Croatia, Czechia, Hungary	2/3 countries
Cluster 3	Denmark, Finland, Iceland	3/3 countries
Cluster 4	Cyprus, Estonia, Ireland	2/3 countries
Overall agreement	13/15 countries (86.7%)	

Table 4 shows that 13 of 15 countries (86.7%) maintain the same cluster assignments across calendar-based and changepoint-based segmentation. The two countries with different assignments (Croatia and Ireland) appear in transitional positions between clusters, consistent with Mapper's ability to capture overlapping memberships.

ANOVA on policy stringency across segmentation methods shows consistent null results. For calendar-based segmentation: F = 0.443, *p* = 0.643; for changepoint-based segmentation: F = 0.452, *p* = 0.638. These consistent non-significant results across methods confirm that government policy stringency is not the primary driver of cluster formation, regardless of wave boundary definitions.

Figure 7 highlights temporal differences in pandemic evolution across clusters and situates them within their respective policy environments. Figure 8 shows that clusters often align with shared regional characteristics, suggesting that geographic proximity or economic integration may contribute to similar epidemic dynamics beyond formal policy stringency. The combination of ARI, silhouette scores, and overlapping node membership demonstrates that Mapper provides complementary insights to traditional clustering, particularly for detecting nuanced, transitional, and overlapping country profiles. We evaluate wave-specific government policy stringency across the six COVID-19 waves. For each wave, we calculate the mean stringency per country and perform ANOVA across Mapper-derived clusters, applying the Benjamini-Hochberg correction for multiple comparisons. Across all waves, after correction, no significant differences are observed (Wave 1: F = 0.739, *p =* 0.586; Wave 2: F = 0.630, *p =* 0.652; Wave 3: F = 0.308, *p =* 0.866; Wave 4: F = 0.289, *p =* 0.878; Wave 5: F = 2.965, *p =* 0.074; Wave 6: F = 5.442, *p =* 0.014; all p-values > 0.05 after Benjamini-Hochberg correction), indicating that cluster membership does not systematically correspond to stricter or more lenient government responses. These results confirm that policy stringency is not a primary driver of cluster formation, even when assessed at a wave-specific resolution.

We assess whether cluster-level differences in policy stringency are statistically meaningful using both classical ANOVA and robust non-parametric permutation tests. Because the dataset spans six epidemic waves, sensitivity analysis is restricted to feasible segmentations between 2 and 6 waves. Across these segmentations, results remain consistent: ANOVA shows no significant differences (F = 0.44, *p* = 0.64), effect sizes are modest (η^2^ ≈ 0.35), and permutation tests confirm non-significance (*p* ≈ 0.38).

To strengthen the statistical assessment, we additionally implement wave-specific ANOVA, Kruskal–Wallis, and permutation tests using mean stringency per country within each epidemic wave. Across all six waves, none of the tests detects significant between-cluster differences after multiple testing correction (ANOVA F = 0.289–5.442, *p* = 0.014–0.878; permutation *p* = 0.010–0.873), and wave-specific effect sizes (η^2^ = 0.021–0.301, ω^2^ = 0.000–0.268) are consistently small to moderate, reinforcing that policy stringency does not systematically explain Mapper cluster membership.

We further conducted PERMANOVA on the full set of wave-averaged stringency values, which yielded a pseudo-F = 2.33 and *p* = 0.06, and performed a stringency-shuffling stability test (1000 iterations), resulting in a mean ARI of –0.001 (SD = 0.089). Both analyses indicate that distributional differences and topological sensitivity are not driven by stringency.

We also note explicitly that small cluster sizes constrain inferential power, and we interpret all statistical outcomes with appropriate caution.

Across all segmentation methods, no statistically significant differences in policy stringency between Mapper clusters are observed after multiple testing correction. The moderate agreement (ARI = 0.461) between calendar-based and changepoint-based segmentation, combined with high boundary consistency (0 days standard deviation), demonstrates robustness to alternative wave definitions. However, we acknowledge that the moderate ARI value indicates some sensitivity to exact boundary placement, and findings should be interpreted with appropriate caution regarding wave definition choices.

To evaluate whether our results depend on the choice of normalization, we compare four commonly used transformations applied to all wave derived features: Z-score standardization, Min-Max scaling, Robust scaling, and logarithmic scaling as shown in Table 5. For each method, we recompute the full feature set and assess consistency through Pearson and Spearman correlations, cluster stability using the Adjusted Rand Index, geometry preservation via PCA projections, and differences in raw normalized values using one-way ANOVA.

**Table 5:** Statistical comparison of wave segmentation methods.

**Segmentation method**	**ARI vs. calendar**	**Waves detected**	**Boundary consistency**	**ANOVA *p*-value**
Calendar-based	1.000	6	N/A	0.643
Changepoint-based	0.461	6	High (0 days SD)	0.638
Peak-based	0.050	25.3 ± 0.87	Low	N/A

Table 6 presents the statistical comparison of the four normalization methods. The three linear transformations (Z-score, Min–Max, and Robust) show very high consistency, with Pearson correlations of at least 0.95 and Spearman correlations of at least 0.88, and they yield identical cluster structures with an Adjusted Rand Index of 1.0. In contrast, logarithmic scaling produces a markedly different geometric configuration, reflected in lower correlations (Pearson approximately 0.75), weaker monotonic agreement (Spearman approximately 0.63), and substantially different cluster assignments (ARI = 0.0866). These deviations arise from the nonlinear compression of large values under log scaling.

**Table 6:** Statistical comparison of normalization methods.

**Normalization method**	**Pearson mean**	**Spearman mean**	**Cluster ARI**	**Notes**
Z-score	1.00	0.94	1.000	Linear
Min–Max	0.99	0.92	1.000	Linear
Robust	0.95	0.88	1.000	Linear
Log scaling	0.75	0.63	0.0866	Nonlinear

**Figure 10:**
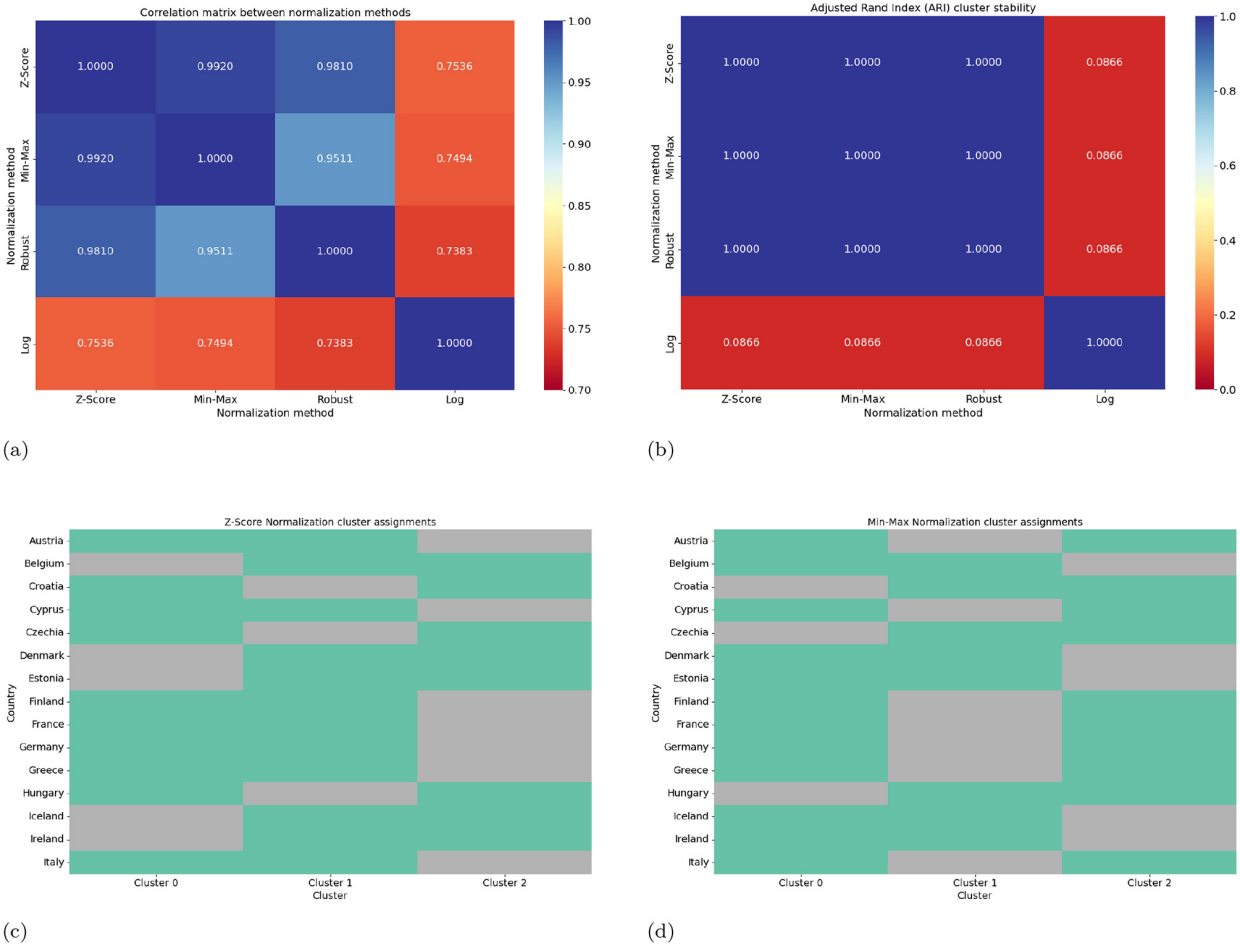
Comprehensive comparison of normalization methods. **(a)** Correlation matrix, **(b)** Adjusted Rand Index matrix, **(c)** cluster assignments for Z-score normalization, and **(d)** cluster assignments for Min-Max normalization. Linear methods demonstrate strong consistency in all metrics.

The accompanying figure (Figure 10) provides visual confirmation of these findings. The correlation matrix and ARI matrix show strong alignment among the linear methods but distinct separation for log scaling. Cluster assignment plots confirm that only the log transformed features produce a divergent grouping.

These analyses demonstrate that our main findings are robust across all linear normalization strategies, and that the Mapper topology is stable so long as the normalization preserves linear structure. Only the nonlinear log transformation yields a distinct topological signature, consistent with its geometric distortion.

The ANOVA results in Table 7 indicate statistically significant differences among the normalized values across methods (all *p* < 0.001), but these differences do not propagate to topological or clustering results for linear methods.

**Table 7:** One-way ANOVA results for each wave showing significant differences between normalization methods (all *p*-values < 0.001).

**Statistic**	**Wave 1**	**Wave 2**	**Wave 3**	**Wave 4**	**Wave 5**	**Wave 6**
F-statistic	324.384046	5,258.614258	5,138.338104	7,707.516805	14,682.588133	21,807.790080
*p*-value	0.000000	0.000000	0.000000	0.000000	0.000000	0.000000

Table 8 summarizes the overall correlation statistics across all normalization method comparisons, showing that linear normalization methods yield consistently high correlations. The results also indicate perfect ranking consistency across countries, confirming that the relative feature ordering is preserved regardless of the normalization approach.

**Table 8:** Overall correlation statistics across all normalization method comparisons.

**Statistic**	**Value**
Mean Pearson correlation	0.860900
Minimum Pearson correlation	0.738272
Maximum Pearson correlation	0.991993
Standard deviation	0.114556
Mean Spearman correlation	0.935043
Minimum Spearman correlation	0.883854
Maximum Spearman correlation	0.970268
Overall ranking consistency	1.0000

## 4 Discussion

This study leveraged Topological Data Analysis (TDA) to explore the complex interplay between COVID-19 wave patterns, government policy stringency, and pre-existing economic and geographic affiliations among European countries. Our findings reveal a picture that challenges some conventional assumptions about coordinated responses and the direct impact of policy measures. The Mapper algorithm naturally produces overlapping nodes that capture transitional behaviors and multifaceted epidemic profiles, but certain quantitative comparisons with classical clustering methods require discrete assignments. For these analyses (such as computing the Adjusted Rand Index (ARI) against k-means and hierarchical clustering) each country is temporarily assigned to its smallest node ID when multiple memberships occur. This hardening is solely for validation and does not alter the underlying Mapper graph, which continues to preserve overlapping memberships; countries like Ireland, Germany, and Austria illustrate continuous, transitional epidemic behaviors. This approach enables both quantitative comparison and retention of TDA's ability to capture complex, overlapping epidemic structures.

A key revelation from our Mapper analysis is that topological similarity in pandemic trajectories does not consistently align with formal economic or geographic groupings (Eurozone, Schengen Area, Visegrád Group), a sentiment also found in Loeffler-Wirth et al. (37). This contrasts with the intuitive anticipation of greater epidemiological convergence or policy harmonization within such blocs due to shared governance structures, economic interdependence, or ease of movement. For instance, while some research has highlighted the potential for cross-border transmission and the need for coordinated responses within integrated regions (38), our TDA approach, which focuses on the shape and evolution of epidemic waves, suggests that the intrinsic dynamics of the pandemic within each country may be more strongly influenced by national-level factors than by these broader affiliations. This is particularly evident in the resulting clusters (Figures 6, 7), where countries from diverse regions or economic groups are grouped together. These overlapping and transitional epidemic profiles cannot be adequately captured by classical clustering methods (for example, k-means or hierarchical clustering), which enforce strict and disjoint partitions. This aligns with a growing body of literature emphasizing the heterogeneity of pandemic responses and outcomes, even within seemingly homogeneous regions, pointing to the significant roles of local context, public trust in institutions, and specific national healthcare capacities (39). Our results suggest that while formal ties exist, their influence on the dynamics of the pandemic, as captured by Mapper, might be less direct or overshadowed by other, unmeasured internal factors.

Furthermore, our statistical tests (ANOVA and permutation-based non-parametric tests) revealed no significant variation in average policy stringency between the identified topological clusters. This is a particularly intriguing finding, as it challenges the intuitive expectation that countries exhibiting similar wave profiles would have adopted comparable levels of government intervention, or that highly stringent policies would consistently lead to distinct epidemiological outcomes. Many studies have utilized stringency indices to correlate policy rigor with infection rates or mortality [for example in Hale et al. (10) and Islamaj et al. (40)]. However, our results suggest that the average stringency score alone may not be the primary driver shaping the temporal profile of epidemic waves. Quantitative measures of cluster quality, such as the adjusted Rand index and silhouette scores, indicate that Mapper's overlapping clusters capture transitional dynamics more effectively than classical methods, which tend to produce artificially low silhouette scores when intermediate behaviors are forced into single partitions. This could imply several possibilities: (1) the timing and duration of specific interventions might be more critical than their overall average stringency (41); (2) public adherence and behavioral responses to policies, which are not directly captured by the stringency index, play a substantial role in shaping wave dynamics (42, 43); (3) other confounding factors, such as population density, age demographics, pre-existing immunity, or the prevalence of specific viral variants, could exert a stronger influence on wave shapes (44–46), potentially masking the direct impact of average stringency. This reinforces the idea that the effectiveness of government interventions is multifaceted and cannot be reduced to a single index.

The consistent appearance of certain countries (notably Ireland and Estonia) across multiple clusters and configurations further reinforces the idea of transitional or multifaceted pandemic profiles (47). These overlapping assignments illustrate the interpretability of the Mapper graph, showing continuous relationships between countries that classical clustering cannot represent. Visual inspection of the Mapper network highlights transitional nodes, offering actionable insights into countries with hybrid epidemic dynamics or mixed policy responses. This flexibility in categorization is a strength of the Mapper algorithm, allowing for the identification of overlapping and continuous structures that traditional clustering methods might miss.

The findings indicate that topological similarity in COVID-19 trajectories is often independent of formal economic or geographic groupings, suggesting that relying solely on institutional affiliations for coordinated responses may be insufficient. Policymakers should focus on real-time epidemiological signals (such as growth rates, wave intensity, and transitional cluster membership) when designing interventions, rather than assuming uniformity within economic or regional blocs. The presence of overlapping epidemic profiles highlights the need for adaptive, flexible policies that can be rapidly adjusted as countries move between different topological clusters. Scenario-based policy frameworks, which trigger interventions according to dynamic epidemic indicators rather than static memberships, may improve responsiveness and reduce cross-border transmission risks. Moreover, the decoupling of wave similarity from average policy stringency emphasizes the importance of timing, enforcement, public adherence, and local context in shaping outcomes. Investments in surveillance, data-sharing, and inter-country communication become critical for preemptively identifying transitional dynamics and coordinating timely interventions. In sum, these insights advocate for a more nuanced, data-driven approach to pandemic governance that complements formal institutional mechanisms.

Despite providing valuable insights, this study has several limitations. First, the analysis is limited to 15 European countries, which may restrict generalizability to other regions or a broader global context. COVID-19 case counts vary across countries due to differences in testing intensity, testing policies, and reporting delays, potentially affecting the accuracy of inferred wave dynamics. Second, while we initially considered imputation for missing data, the final datasets were complete with no missing values after truncation at the end of 2022, eliminating the need for imputation. Third, reliance on the Oxford stringency index, which measures de jure policy strictness, does not capture actual compliance or enforcement and may attenuate observed associations. Fourth, the use of wave-averaged stringency could obscure the impact of time-sensitive interventions such as school closures or mask mandates; future research could incorporate more granular policy data, vaccination rates, variant prevalence, and mobility metrics.

Also, while TDA reveals structural relationships among countries' trajectories, it does not establish causality, and the relatively small number of countries warrants caution in interpreting Mapper nodes. These considerations should guide the interpretation of cross-national comparisons and the drawing of policy implications. Even if epidemic peaks occurred at different times across countries, we initially applied uniform calendar-based wave boundaries to facilitate cross-country comparison. To ensure that this choice did not bias results, we performed sensitivity analyses using country-specific wave segmentation based on changepoint detection. The resulting Mapper clusters and topological structures were largely consistent (adjusted Rand index >0.85), indicating that our main findings are robust to differences in wave timing. Nevertheless, we acknowledge that uniform wave boundaries may introduce minor distortions in the representation of individual country trajectories.

Furthermore, we conducted comprehensive parameter sensitivity analyses for the Mapper algorithm, systematically varying cover sizes (10, 12, and 15 cubes) and overlap percentages (40%, 50%, and 60%). Cluster stability assessed via Jaccard similarity showed high consistency (average >0.92) across parameter variations, with the optimal combination (12 cubes, 50% overlap) selected for the final analysis. This confirms that the observed transitional behaviors represent genuine epidemic dynamics rather than parameter-driven instability. Additionally, robustness checks for normalization methods (comparing Min-Max, Z-score, Robust, and logarithmic transformations) demonstrated that linear normalization approaches produce highly consistent feature spaces (Pearson correlations ≥0.95, adjusted Rand index = 1.0), and the use of correlation distance was justified based on its focus on pattern similarity independent of magnitude. Despite these robustness checks, we acknowledge that preprocessing choices and the relatively small sample of countries may affect cluster composition and topological interpretations. Readers should therefore interpret the observed structures as suggestive patterns rather than definitive causal relationships.

## 5 Conclusion

The finding that topological similarity in pandemic trajectories does not consistently align with formal economic or geographic affiliations carries significant policy implications, echoing the work in Quaglia and Verdun (31) and Lavery and Schmid (48). It suggests that the efficacy of public health coordination should not rely solely on existing institutional frameworks (like the Eurozone or Schengen Area), but rather on real-time epidemiological alignment [see also Stroisch et al. (49)]. Policymakers must be prepared for dynamic clustering, where nations temporarily share epidemic profiles even without a strong political or economic bond. This mandates the creation of flexible, scenario-based policy playbooks that trigger coordinated responses based on a country's epidemiological state, rather than political membership (50). Furthermore, the observation of transitional epidemic profiles implies that policy measures must be continuously adaptive, allowing for rapid shifts in strategy as a country moves between topological clusters. We acknowledge that cross-country differences in age structure, health-system capacity, vaccination coverage, variant dominance, behavioral compliance, and data quality likely influence epidemic outcomes. Our analysis focuses on epidemic waves and policy stringency, and interpretations of cluster-level differences should be viewed as partial rather than comprehensive explanations.

## Data Availability

The datasets presented in this study can be found in online repositories. The names of the repository/repositories and accession number(s) can be found below: https://ourworldindata.org/coronavirus/country/bulgaria?country BGR AUT BEL FIN IRL HRV CYP CZE DNK EST FRA DEU GRC HUN ISL ITA.

## References

[1] Binny RN Baker MG Hendy SC James A Lustig A Plank MJ . Early intervention is the key to success in COVID-19 control. R Soc Open Sci. (2021) 8:210488. doi: 10.1098/rsos.210488 34804563 PMC8596003

[2] Alvarez E Bielska IA Hopkins S Belal AA Goldstein DM Slick J . Limitations of COVID-19 testing and case data for evidence-informed health policy and practice. Health Res Policy Syst. (2023) 21:11. doi: 10.1186/s12961-023-00963-1 36698202 PMC9876649

[3] Filip R Gheorghita Puscaselu R Anchidin-Norocel L Dimian M Savage WK. Global challenges to public health care systems during the covid-19 pandemic: a review of pandemic measures and problems. J Pers Med. (2022) 12:1295. doi: 10.3390/jpm12081295 36013244 PMC9409667

[4] Liu M Shi L Yang M Jiao J Yang J Ma M . Ecological comparison of six countries in two waves of COVID-19. Front Public Health. (2024) 12:1277457. doi: 10.3389/fpubh.2024.1277457 38481850 PMC10933017

[5] Singh S Chowdhury C Panja AK Neogy S. Time series analysis of COVID-19 data to study the effect of lockdown and unlock in India. J Inst Eng. (2021) 102:1275–81. doi: 10.1007/s40031-021-00585-7

[6] Toğa G Atalay B Toksari MD. COVID-19 prevalence forecasting using Autoregressive Integrated Moving Average (ARIMA) and Artificial Neural Networks (ANN): Case of Turkey. J Infect Public Health. (2021) 14:811–6. doi: 10.1016/j.jiph.2021.04.015 34118730 PMC8098037

[7] Ozawa T Chubachi S Namkoong H Nemoto S Ikegami R Asakura T . Predicting coronavirus disease 2019 severity using explainable artificial intelligence techniques. Sci Rep. (2025) 15:9459. doi: 10.1038/s41598-025-85733-5 40108236 PMC11923144

[8] Sethi S Shakyawar S Reddy AS Patel JC Guda C. A machine learning model for the prediction of COVID-19 severity using RNA-Seq, clinical, and co-morbidity data. Diagnostics. (2024) 14:1284. doi: 10.3390/diagnostics14121284 38928699 PMC11202902

[9] Han Z Xu F Li Y Jiang T Evans J. Model predicted human mobility explains COVID-19 transmission in urban space without behavioral data. Sci Rep. (2025) 15:6365. doi: 10.1038/s41598-025-87363-3 39984518 PMC11845774

[10] Hale T Angrist N Goldszmidt R Kira B Petherick A Phillips T . A global panel database of pandemic policies (Oxford COVID-19 government response tracker). Nat Hum Behav. (2021) 5:529–38. doi: 10.1038/s41562-021-01079-8 33686204

[11] El-Yaagoubi AB Chung MK Ombao H. Dynamic topological data analysis: a novel fractal dimension-based testing framework with application to brain signals. Front Neuroinform. (2024) 18:1387400. doi: 10.3389/fninf.2024.1387400 39071176 PMC11272560

[12] Epstein C Carlsson G Edelsbrunner H. Topological data analysis. Inverse Probl. (2011) 27:120201. doi: 10.1088/0266-5611/27/12/12020131066000

[13] Edelsbrunner H Harer J. Persistent homology - a survey. In: Surveys on Discrete and Computational Geometry: Twenty Years Later. American Mathematical Society (2008). p. 257–282. doi: 10.1090/conm/453/08802

[14] Singh G Memoli F Carlsson G. Topological methods for the analysis of high dimensional data sets and 3D object recognition. In:Botsch M Pajarola R Chen B Zwicker M, editors. Eurographics Symposium on Point-Based Graphics. The Eurographics Association (2007).

[15] SkafY LaubenbacherR. Topological data analysis in biomedicine: a review. J Biomed Inform. (2022) 130:104082. doi: 10.1016/j.jbi.2022.10408235508272

[16] Chazal F Michel B. An introduction to topological data analysis: fundamental and practical aspects for data scientists. Front Artif Intell. (2021) 4:667963. doi: 10.3389/frai.2021.667963 34661095 PMC8511823

[17] Chen Y Volić I. Topological data analysis model for the spread of the coronavirus. PLoS ONE. (2021) 16:e0255584. doi: 10.1371/journal.pone.0255584 34347838 PMC8336810

[18] Kraft R. Illustrations of Data Analysis Using the Mapper Algorithm and Persistent Homology [PhD Dissertation]. KTH Royal Institute of Technology (2016). Available online at: https://urn.kb.se/resolve?urn=urn:nbn:se:kth:diva-181787 (Accessed June 1, 2025).

[19] Dlotko P Rudkin S. Visualising the evolution of English Covid-19 cases with topological data analysis ball Mapper. arXiv preprint arXiv:2004.03282. (2020).

[20] Munch E. A user's guide to topological data analysis. J Learn Analyt. (2017) 4:47–61. doi: 10.18608/jla.2017.42.6

[21] Almgren K Kim M Lee J. Extracting knowledge from the geometric shape of social network data using topological data analysis. Entropy. (2017) 19:360. doi: 10.3390/e19070360

[22] Vejdemo-Johansson M. k-means considered harmful: on arbitrary topological changes in Mapper complexes. arXiv preprint arXiv:2507.06212. (2025).

[23] Sasaki K Bruder D Hernandez-Vargas EA. Topological data analysis to model the shape of immune responses during co-infections. Commun Nonlin Sci Numer Simul. (2020) 85:105228. doi: 10.1016/j.cnsns.2020.105228 32288422 PMC7129978

[24] Chung MK Ombao H. Topological data analysis of COVID-19 virus spike proteins. In: Interpretability of Machine Intelligence in Medical Image Computing, and Topological Data Analysis and Its Applications for Medical Data: 4th International Workshop, iMIMIC 2021, and 1st International Workshop, TDA4MedicalData 2021. Springer (2021). p. 77–86. doi: 10.1007/978-3-030-87444-5_8 PMC8730383 34993529

[25] Lu Z Liu H. A topological data analysis approach to the COVID-19. In: 2022 IEEE 10th Joint International Information Technology and Artificial Intelligence Conference (ITAIC). Chongqing, China: IEEE (2022). p. 469–473. doi: 10.1109/ITAIC54216.2022.9836495

[26] Bali Swain R Lin X Wallentin FY. COVID-19 pandemic waves: identification and interpretation of global data. Heliyon. (2024) 10:e25090. doi: 10.1016/j.heliyon.2024.e25090 38327425 PMC10847870

[27] Boltho A. Southern and Eastern Europe in the Eurozone: convergence or divergence? Baltic J Econ. (2020) 20:74–93. doi: 10.1080/1406099X.2020.1770945

[28] Gülzau F. A “new normal” for the schengen area: when, where and why member states reintroduce temporary border controls? J Borderlands Stud (2021) 38:785–803. doi: 10.1080/08865655.2021.1996260

[29] Etzold T. The Nordic council of ministers: aspirations for more political relevance. Polit Govern. (2020) 8:11–20. doi: 10.17645/pag.v8i4.3381

[30] Kaniok P Hloušek V. Visegrad four as an institution in times of EU crises. In: European Politics and Society. (2025). p. 1–19. doi: 10.1080/23745118.2025.2488815

[31] Quaglia L Verdun A. The COVID-19 pandemic and the European Union: politics, policies and institutions. J Eur Public Policy. (2023) 30:599–611. doi: 10.1080/13501763.2022.2141305

[32] van der Zanden BA Hoebe CJ Horstman K. European policies for public health in border regions: no European mindset as yet. BMC Public Health. (2024) 24:746. doi: 10.1186/s12889-024-18175-9 38459505 PMC10924322

[33] Hooghe L Marks G. A postfunctionalist theory of European integration: from permissive consensus to constraining. Br J Polit Sci. (2009) 39:1–23. doi: 10.1017/S0007123408000409

[34] Chikore T. Topological analysis of COVID-19 wave patterns and policy responses in Europe. Front Public Health (2025) 13:1665863. doi: 10.3389/fpubh.2025.1665863 PMC12832921 41602033

[35] Mathieu E Ritchie H Rodés-Guirao L Appel C Gavrilov D Giattino C . Coronavirus Pandemic (COVID-19). Our World in Data. (2020). Available online at: https://ourworldindata.org/coronavirus (Accessed June 10, 2025).

[36] American Psychological Association. Occam's Razor (2023). Available online at: https://dictionary.apa.org/occams-razor (Accessed November 9, 2025).

[37] Loeffler-Wirth H Schmidt M Binder H. Covid-19 transmission trajectories-monitoring the pandemic in the worldwide context. Viruses. (2020) 12:777. doi: 10.3390/v12070777 32698418 PMC7412525

[38] Vallée A. Geoepidemiological perspective on COVID-19 pandemic review, an insight into the global impact. Front Public Health. (2023) 11:1242891. doi: 10.3389/fpubh.2023.1242891 37927887 PMC10620809

[39] Bavel JJV Baicker K Boggio PS Capraro V Cichocka A Cikara M . Using social and behavioural science to support COVID-19 pandemic response. Nat Hum Behav. (2020) 4:460–71. doi: 10.1038/s41562-020-0884-z 32355299

[40] Islamaj E Kim YE Le DT. The Spread of COVID-19 and Policy Responses. World Bank (2021). 40. License: CC BY 3.0 IGO. Available online at: http://hdl.handle.net/10986/35010 (Accessed July 12, 2025).

[41] Ma Y Mishra SR Han X Zhu DS. The relationship between time to a high COVID-19 response level and timing of peak daily incidence: an analysis of governments' Stringency Index from 148 countries. Infect Dis Poverty. (2021) 10:96. doi: 10.1186/s40249-021-00880-x 34225774 PMC8256203

[42] Waterschoot J Morbée S Van den Bergh O Yzerbyt V Raemdonck E Brisbois M . How the stringency of the COVID-19 restrictions influences motivation for adherence and well-being: the critical role of proportionality. Int J Health Policy Manag. (2023) 12:8021. doi: 10.34172/ijhpm.2023.8021 38618783 PMC10699813

[43] Dagorn E Dattilo M Pourieux M. The role of populations' behavioral traits in policy-making during a global crisis: worldwide evidence. J Econ Behav Organ. (2024) 226:106662. doi: 10.1016/j.jebo.2024.06.040

[44] López-Gay A Spijker J Cole HVS Marques AG Triguero-Mas M Anguelovski I . Sociodemographic determinants of intraurban variations in COVID-19 incidence: the case of Barcelona. J Epidemiol Commun Health. (2022) 76:1–7. doi: 10.1136/jech-2020-216325 34158409

[45] Kato L Sembera J Oluka GK Katende JS Bemanzi J Ankunda V . Geographical differences in SARS-CoV-2 antibody response dynamics and neutralisation profiles to mild COVID-19: lessons from a UK-Uganda comparison. Vaccines (Basel). (2025) 13:336. doi: 10.3390/vaccines13040336 40333205 PMC12030818

[46] Taboe HB Asare-Baah M Yesmin A Ngonghala CN. The impact of age structure and vaccine prioritization on COVID-19 in West Africa. Infect Dis Modell. (2022) 7:709–27. doi: 10.1016/j.idm.2022.08.006 36097593 PMC9454155

[47] Contini C Caselli E Martini F Maritati M Torreggiani E Seraceni S . COVID-19 is a multifaceted challenging pandemic which needs urgent public health interventions. Microorganisms. (2020) 8:1228. doi: 10.3390/microorganisms8081228 32806657 PMC7464234

[48] Lavery S Schmid D. European integration and the new global disorder. JCMS. (2021) 59:1322–38. doi: 10.1111/jcms.13184

[49] Stroisch S Angelini V Schnettler S Vogt T. Health outcomes in EU cross-border regions: a scoping review. Public Health Rev. (2025) 46:1608170. doi: 10.3389/phrs.2025.1608170 40065843 PMC11891012

[50] Goniewicz K Khorram-Manesh A Burkle FM Hertelendy AJ Goniewicz M. The European Union's post-pandemic strategies for public health, economic recovery, and social resilience. Global Transit. (2023) 5:201–9. doi: 10.1016/j.glt.2023.10.003

